# Clinical features and outcomes of herpes simplex viral infections with co-infection in 100 children

**DOI:** 10.3389/fped.2026.1774544

**Published:** 2026-04-15

**Authors:** Ping Wang, Jian Li

**Affiliations:** Infectious Diseases Department, Jinan Children's Hospital (Children's Hospital Affiliated to Shandong University), Jinan, Shandong, China

**Keywords:** children, clinical features, fever, herpes simplex virus, treatment

## Abstract

**Objective:**

To analyze the clinical features, laboratory findings, and treatment outcomes of febrile hospitalized children with herpes simplex virus (HSV) detected by serological and/or molecular biological tests, and to provide an evidence-based basis for the early clinical screening, differential diagnosis and targeted intervention of HSV in pediatric febrile cases.

**Methods:**

A retrospective analysis was performed on the clinical data of 100 febrile children with positive HSV test results who were admitted to Jinan Children's Hospital from January 2018 to October 2024. The collected data included demographic characteristics, clinical manifestations, confirmed clinical diagnoses, co-detected pathogen status(diagnosed by a combination of serology, PCR and microbial culture), laboratory examination indicators, treatment regimens(including individualized acyclovir course), and prognostic outcomes. HSV detection was conducted by serum HSV-IgM antibody assay and/or HSV-DNA detection in pharyngeal swab/blood samples, and the number of cases with single positive and double positive results of the two tests was statistically analyzed separately. All statistical analyses were performed using SPSS 26.0 software, with measurement data described as median [interquartile range (IQR)] and compared by Mann–Whitney U test. A two-sided *P* < 0.05 was considered statistically significant.

**Results:**

Among the 100 children, 62 were male and 38 were female, with a male-to-female ratio of 1.6:1. The age ranged from 1 month to 8 years, with a median age of 2.1 years, and the 1 to <3 years age group accounted for the highest proportion (56.0%, 56/100). Fever was the primary admission symptom in all cases, with 72.0% (72/100) presenting with high fever (≥39 °C). The most common local manifestation was oral mucosal lesions (19.0%, 19/100). Physical examination showed cervical lymphadenopathy in 85.0% (85/100) and pharyngeal mucosal congestion/redness in 92.0% (92/100) of the children. Of the 100 HSV-positive cases, 65 (65.0%) were double positive for HSV-IgM and HSV-DNA, 23 (23.0%) were single positive for HSV-IgM, and 12 (12.0%) were single positive for HSV-DNA. Other pathogens were co-detected in 52.0% (52/100) of the cases, with Epstein–Barr virus (EBV) being the most common (27.0%, 27/100). Abnormal liver function (elevated alanine aminotransferase [ALT] and/or aspartate aminotransferase [AST]) was found in 30.0% (30/100) of the children. Ninety-two children received intravenous acyclovir for antiviral treatment, with a median time to defervescence of 2.5 days (IQR: 2.0–3.0) and a median hospital stay of 7.0 days (IQR: 6.0–8.0). All children were cured and discharged. Statistical analysis showed that children who received early acyclovir treatment (within 24 h of admission) had significantly shorter time to defervescence and hospital stay compared with those in the delayed treatment group (>24 h) (Z = −3.874 and −4.125, respectively, both *P* < 0.001). Co-detection of EBV and abnormal liver function were associated with a significant prolongation of hospital stay (Z = −3.987 and −4.563, respectively, both *P* < 0.001).

**Conclusion:**

HSV is a frequently isolated pathogen in febrile infants and young children with herpetic gingivostomatitis as the core clinical diagnosis and diverse clinical manifestations dominated by non-specific systemic and oropharyngeal symptoms (cutaneous herpes with low incidence). HSV infection is often accompanied by co-detection of other pathogens such as EBV and Bordetella pertussis (diagnosed by standardized serological, PCR and microbial culture methods) and abnormal liver function in some cases. Early HSV-PCR detection combined with serological testing, timely and individualized acyclovir treatment (with clear course standards) based on comprehensive clinical judgment can significantly improve the clinical outcomes of febrile children with HSV detected. Comprehensive intervention targeting co-detected pathogens and complications is equally important for the clinical management of such cases.

## 1 Introduction

Fever is one of the most common clinical complaints in pediatric patients, accounting for nearly one-third of global pediatric outpatient visits (1). Infections are the most frequent cause of fever of unknown origin in all pediatric studies, including bacterial infections, brucellosis, tuberculosis, and typhoid fever. Viral infections, particularly herpesvirus infections, are also a significant cause (2). As a member of the Herpesviridae family, herpes simplex virus (HSV) is a common pathogenic virus in the pediatric population, which can cause mucocutaneous lesions such as stomatitis and skin herpes, and even life-threatening systemic infections in severe cases (3). Due to the immature immune system and incomplete development of mucosal barriers, infants and young children are more susceptible to HSV infection, and the infected children are prone to develop high fever and feeding difficulties, often requiring hospitalization for systematic treatment.

In recent years, with the popularization of detection techniques, the detection rate of HSV in febrile children has increased. However, its clinical features can easily be confused with other viral infections (such as EBV, enteroviruses), leading to delayed treatment. Globally, approximately 67% of the population is infected with HSV-1, and the seroprevalence in children under 5 years old in developing countries can reach 80% (4). The HSV-1 infection rate in Chinese adults exceeds 90%, but data in children are limited, especially the clinical data of hospitalized febrile children with clear clinical diagnosis, standardized co-infection detection methods and definite acyclovir treatment courses. This study fills this gap by analyzing the characteristics of HSV infection in hospitalized febrile children. Currently, there are few systematic analyses of HSV infection in hospitalized febrile children in China, particularly lacking data on co-infections and treatment outcomes. This retrospective study analyzed the clinical data of 100 children with positive HSV, aiming to summarize its clinical and therapeutic characteristics and provide references for early identification and precise intervention.

## 2 Materials and methods

### 2.1 Study subjects

A retrospective cohort study was conducted on febrile children admitted to Jinan Children's Hospital from January 2018 to October 2024. The inclusion criteria were formulated as follows: (1) Age ≤14 years; (2) Febrile symptoms with rectal temperature ≥38 °C or axillary temperature ≥37.3 °C; (3) Positive results of serum HSV-IgM antibody detection and/or HSV-DNA detection in pharyngeal swab/blood samples. The exclusion criteria were: (1) Children with congenital immunodeficiency diseases confirmed by clinical and laboratory examinations; (2) Children complicated with severe underlying diseases, including congenital heart disease, chronic liver and kidney insufficiency, malignant tumors, etc.; which may affect the clinical manifestations and treatment outcomes of HSV infection; (3) Children with incomplete clinical data that cannot be used for statistical analysis. A total of 100 children who met the above criteria were enrolled in this study, all of whom were healthy without any underlying diseases or comorbidities.

### 2.2 Methods

#### 2.2.1 Data collection

Clinical data were collected comprehensively from the hospital's electronic medical record system, including: (1) Demographic characteristics: gender, age grouping; (2) Clinical manifestations: duration of fever before admission, peak temperature, oral and mucocutaneous manifestations, convulsions, respiratory symptoms and other systemic symptoms; (3) Confirmed clinical diagnoses: core diagnosis, comorbid diagnoses and diagnostic basis based on clinical symptoms, physical signs, etiological test results and auxiliary examinations; (4) Laboratory indicators: complete blood count, liver function, inflammatory markers (CRP), etiological test results; (5) Treatment and prognostic outcomes: use of antiviral drugs, dosage, administration route and individualized course of acyclovir, targeted treatment for co-infections and complications, time to defervescence, length of hospital stay, curative effect and adverse reactions.

#### 2.2.2 Etiological detection methods

(1) HSV detection: ① Serological testing: Serum HSV-IgM antibody was detected by enzyme-linked immunosorbent assay (ELISA) using the HSV-IgM ELISA Kit (Cat. No. E-Human0012, Elabscience Biotechnology Co., Ltd., Wuhan, China), and the optical density (OD) value was measured at 450 nm by a microplate reader (Model Multiskan FC, Thermo Fisher Scientific, USA); ② Molecular biological testing: Quantitative fluorescent PCR was used to detect HSV-DNA in pharyngeal swab or blood samples.
(2) Co-pathogen detection(diagnosed by pathogen-specific standardized methods, with clear kits and instruments for all detections):

① EBV detection: EBV viral capsid antigen (VCA)-IgM antibody was detected by ELISA (Cat. No. E-Human0015, Elabscience Biotechnology Co., Ltd., Wuhan, China);

② Bacterial detection: Venous blood samples were collected for blood culture using the BACTEC FX blood culture system (BD Biosciences, USA), and the isolated bacteria were identified by the VITEK 2 Compact automatic microbial identification system (bioMérieux, France);

③ Atypical pathogen detection: Mycoplasma pneumoniae DNA was detected by quantitative fluorescent PCR using the corresponding detection kit (Cat. No. PG-21008, Zhuhai Primgene Biotech Co., Ltd., Zhuhai, China) on the ABI 7500 quantitative PCR instrument.

④ Other viral co-pathogens: Enterovirus, influenza virus, human metapneumovirus, cytomegalovirus, mumps virus and varicella-zoster virus were detected by corresponding quantitative fluorescent PCR or ELISA serological tests based on their biological characteristics.

#### 2.2.3 Clinical diagnosis criteria

All clinical diagnoses were made by senior specialists in the Department of Infectious Diseases according to the Clinical Guidelines for the Diagnosis and Treatment of Pediatric Infectious Diseases and Clinical Diagnosis and Treatment Norms for Herpetic Virus Infections in Children, with comprehensive judgment based on clinical symptoms, physical signs, etiological test results and auxiliary examinations:

(1) Herpetic gingivostomatitis (core diagnosis): Defined by fever combined with typical oropharyngeal manifestations (pharyngeal congestion/redness, oral mucosal herpes/ulcers, cervical lymphadenopathy), positive HSV-IgM and/or HSV-DNA test results, and exclusion of other pathogens causing oral mucosal lesions (e.g., enterovirus, Coxsackievirus);

(2) Acute upper respiratory tract infection: Defined by fever accompanied by cough, runny nose, sore throat and other upper respiratory symptoms, combined with lymphocyte-dominant changes in complete blood count and typical pharyngeal physical signs;

(3) Mild pertussis: Defined by positive Bordetella pertussis in blood culture, combined with typical clinical symptoms including paroxysmal spasmodic cough and cockcrow-like echo, without severe complications such as cough apnea;

(4) Acute pharyngitis/tonsillitis: Defined by positive Streptococcus pyogenes in blood culture, combined with sore throat, tonsillar enlargement with exudate and other local manifestations;

(5) Mild mycoplasmal pneumonia: Defined by positive Mycoplasma pneumoniae DNA, combined with mild cough and no obvious abnormal lung imaging findings.

#### 2.2.4 Statistical analysis

All statistical analyses were performed using the SPSS 26.0 statistical software (IBM Corp., Armonk, USA). Measurement data were tested for normality, and the results showed non-normal distribution; thus, they were described as median (M) and interquartile range (IQR). The Mann–Whitney U test was used for the comparison of measurement data between two groups, and the test statistic (Z value) and exact *P* value were reported. A two-sided *P* value < 0.05 was considered statistically significant.

## 3 Results

### 3.1 General information and clinical diagnosis

A total of 100 children meeting the inclusion criteria were included in the final analysis, all of whom were healthy without congenital immunodeficiency diseases, severe underlying diseases, or other comorbidities that may affect the clinical manifestations and treatment of HSV infection. Among them, 62 (62.0%) were male and 38 (38.0%) were female, with a male-to-female ratio of 1.6:1. Ages ranged from 1 month to 8 years, with a median age of 2.1 years. There were 18 cases (18.0%) aged <1 year, 56 cases (56.0%) aged 1∼<3 years, and 26 cases (26.0%) aged ≥3 years, with the 1 to <3 years age group accounting for the highest proportion.

The core clinical diagnosis of all 100 cases was herpetic gingivostomatitis, and comorbid clinical diagnoses were made according to the type of co-infecting pathogens and additional clinical symptoms (no cases of herpes viral encephalitis or disseminated HSV infection were diagnosed). The detailed distribution of comorbid diagnoses was as follows:

(1) 85 cases (85.0%) complicated with acute upper respiratory tract infection: accompanied by cough, runny nose and other mild upper respiratory symptoms, with lymphocyte-dominant blood routine changes;

(2) 4 cases (4.0%) complicated with mild pertussis: Bordetella pertussis positive by blood culture, with typical paroxysmal spasmodic cough and cockcrow-like echo;

(3) 6 cases (6.0%) complicated with acute pharyngitis/tonsillitis: Streptococcus pyogenes positive by blood culture, with tonsillar enlargement and pharyngeal exudate;

(4) 3 cases (3.0%) complicated with acute bronchitis: Haemophilus influenzae type b positive by blood culture, with mild cough and expectoration;

(5) 2 cases (2.0%) complicated with mild mycoplasmal pneumonia: Mycoplasma pneumoniae DNA positive, with mild dry cough and no obvious lung imaging abnormalities;

No severe complications (e.g., central nervous system involvement, multiple organ failure, septic shock) or fatal cases were found in all enrolled children during hospitalization.

### 3.2 Clinical manifestations

Fever was the primary admission symptom in all 100 children, Among them, 72 cases (72.0%) had a body temperature ≥39.0 °C. The most common local clinical manifestation was oral mucosal herpes/ulcers (19 cases, 19.0%), followed by skin herpes (3 cases, 3.0%), showing a low incidence of typical skin manifestations, all of which were febrile convulsions (occurring at body temperature ≥39 °C, rapid recovery of consciousness after attack, no neurological sequelae). The 4 cases with mild pertussis all presented with typical paroxysmal spasmodic cough and cockcrow-like echo, without cough apnea or other severe symptoms.

Among systemic manifestations, seizures occurred in 5 cases (5.0%). Physical examination showed cervical lymphadenopathy in 85 cases (85.0%) and pharyngeal mucosal congestion or redness in 92 cases (92.0%). The main clinical manifestations is shown in Table 1.

**Table 1:** Clinical manifestations in 100 febrile children with HSV infection [*n* (%)].

Clinical Symptom	Number**(n)**	Percentage**(%)**
Systemic Symptoms		
High Fever（≥39℃）	72	72.0
Seizures	5	5.0
Local Symptoms		
Oral Mucosal Herpes/Ulcers	19	19.0
Skin Herpes	3	3.0
Physical Signs		
Cervical Lymphadenopathy	85	85.0
Pharyngeal Mucosal Congestion/Redness	92	92.0

### 3.3 HSV detection results by different methods

In the 100 febrile children with herpes simplex virus (HSV) detected, 65 cases (65.0%) were double positive for serum HSV-IgM antibody and HSV-DNA (including 45 cases from pharyngeal swab samples and 20 cases from blood samples), 23 cases (23.0%) were single positive for serum HSV-IgM antibody alone, and 12 cases (12.0%) were single positive for HSV-DNA alone (including 8 cases from pharyngeal swab samples and 4 cases from peripheral blood samples). The detailed distribution of HSV detection results is shown in Table 2.

**Table 2:** Distribution of HSV detection results (by test method and sample source, *n* = 100).

HSV Detection Result	Sample Source	Positive Number (n)	Percentage (%)
Serum HSV-IgM (+) only	Serum	23	23.0
HSV-DNA (+) only	Pharyngeal swab: 8 cases; Peripheral blood: 4 cases	12	12.0
Serum HSV-IgM (+) + HSV-DNA (+)	Pharyngeal swab: 45 cases; Blood: 20 cases	65	65.0
Total	—	100	100.0

### 3.4 Co-infections and complications

Other pathogens were co-detected in 52 cases (52.0%) of the 100 children with HSV detected. Viral co-detections were the most common type, accounting for 36.0% (36/100), with EBV being the predominant viral co-pathogen (27.0%, 27/100). Bacterial co-detections were found in 14 cases (14.0%), and Streptococcus pyogenes was the most frequent bacterial pathogen (6.0%, 6/100). Atypical pathogen co-detections were identified in 2 cases (2.0%).

Abnormal liver function was the main complication in children with HSV detected, which was observed in 30 cases (30.0%), manifested as elevated ALT and/or AST. The median level of ALT was 19.5 U/L (IQR: 12.0∼39.0 U/L), and the median level of AST was 38.0 U/L (IQR: 32.0∼45.5 U/L). The distribution of co-detected pathogens is shown in Table 3.

**Table 3:** Distribution of Co-infecting pathogens in 100 children with HSV infection [*n* (%)].

Pathogen Type	Number	Percentage **(%)**
Virus	36	36.0
EB Virus	27	27.0
Enterovirus	3	3.0
Influenza Virus	2	2.0
Other Viruses^a^	4	4.0
Bacteria	14	14.0
Streptococcus pyogenes	6	6.0
Bordetella pertussis	4	4.0
Haemophilus influenzae type b	3	3.0
Other Bacteria^b^	1	1.0
Atypical Pathogens	2	2.0

a Other viruses include 1 case each of human metapneumovirus, cytomegalovirus, mumps virus, and varicella-zoster virus.

b Other bacteria refers to 1 case of Brucella.

### 3.5 Laboratory findings

The key laboratory indicators of the 100 children were all described as median (IQR).White blood cell count (WBC) was 8.11 × 10⁹/L (5.66 ∼10.91) × 10⁹/L, lymphocyte percentage (L%) was 55.9% (43.9∼67.0%), C-reactive protein (CRP) was 1.93 mg/L (0.50∼8.65 mg/L), and platelet count (PLT) was 323 × 10⁹/L (254∼392) × 10⁹/L.The key laboratory indicators of the 100 children were all described as median (IQR).

### 3.6 Treatment and outcomes

All 100 children received targeted antiviral treatment for HSV, with the administration route of acyclovir selected according to the clinical status of the children: 92 children received intravenous acyclovir at a dosage of 20 mg·kg^−1^·d^−1^, divided into 2–3 times; the remaining 8 children with mild symptoms, good oral tolerance and no obvious oral mucosal lesions received oral acyclovir at the same dosage. The selection of intravenous administration was based on the clinical status of the children: most were infants and young children with poor oral tolerance due to oral mucosal lesions and persistent high fever, and intravenous administration could ensure the effective blood drug concentration.

Individualized acyclovir treatment courses were formulated according to the presence of co-infections and liver function status, in strict accordance with the Clinical Guidelines for the Diagnosis and Treatment of Herpetic Virus Infections in Children:

(1) Mild cases: 60 children without co-infections and with normal liver function received acyclovir (intravenous/oral) for 7–10 days to achieve complete viral clearance and reduce the risk of infection recurrence;

(2) Complex cases: 40 children with co-infections (e.g., EBV, Bordetella pertussis) or abnormal liver function received an extended course of 10–14 days, with the course dynamically adjusted according to the recovery of body temperature, clinical symptoms and liver function indicators;

(3) Sequential therapy: For children initially treated with intravenous acyclovir, the administration route was switched to oral acyclovir to complete the remaining course after the body temperature returned to normal and clinical symptoms were significantly relieved (generally 3–5 days), with the total course still following the above standards for mild or complex cases.

During the treatment period, all children received routine hydration and renal function monitoring, and no acyclovir-related adverse reactions such as nephrotoxicity, gastrointestinal reactions or allergic reactions were found. For children with bacterial co-detections (e.g., Bordetella pertussis, Streptococcus pyogenes), targeted antibacterial therapy was added based on drug sensitivity test results (e.g., macrolides for pertussis); for children with abnormal liver function, liver-protecting drugs were given for symptomatic treatment; for children with other viral co-infections, supportive treatment was given according to clinical symptoms.

The median time to defervescence in all treated children was 2.5 days (IQR: 2.0∼3.0 days). All enrolled children were successfully cured and discharged from the hospital, with a median hospital stay of 7.0 days (IQR: 6.0∼8.0 days).

The Mann–Whitney U test was used for intergroup comparison, and the results showed that children in the early treatment group (acyclovir administered within 24 h of admission) had significantly shorter time to defervescence and hospital stay compared with those in the delayed treatment group (acyclovir administered more than 24 h after admission) (Z = −3.874 and Z = −4.125, respectively; both *P* < 0.001). In addition, children with EBV co-detected and abnormal liver function had a significantly prolonged hospital stay compared with those without the above conditions (Z = −3.987 and Z = −4.563, respectively; both *P* < 0.001). The detailed comparison of treatment and outcomes in different groups is shown in Table 4.

**Table 4:** Comparison of treatment and outcomes in HSV-infected children with different characteristics [M(IQR)].

Group Characteristic	Number	Time to Defervescence (days)	Z-value	*P*-value	Hospital Stay (days)
**Treatment Timing**			−3.874	<0.001	
Early Treatment Group(<24 h)	65	2.5 (2.0∼3.0)			6.0 (5.0∼7.0)
Delayed Treatment Group(>24 h)	35	3.5 (3.0∼4.5)			8.0 (7.0∼9.5)
**EBV Co-infection**			−3.521	<0.001	
Present	27	3.5 (3.0∼4.5)			8.0 (7.0∼9.0)
Absent	73	2.5 (2.0∼3.0)			6.0 (5.0∼7.0)
**Liver Function**			−4.326	<0.001	
Abnormal (ALT/AST ↑)	30	4.0 (3.5∼5.0)			9.0 (8.0∼10.0)
Normal	70	2.5 (2.0∼3.0)			6.0 (5.0∼7.0)

## 4 Discussion

This single-center retrospective study analyzed the clinical data of 100 febrile hospitalized children with HSV detected by serological and/or molecular biological tests, and found that infants and young children were the main population with HSV detected, with a high rate of co-detected pathogens (especially EBV), and liver function impairment was a common complication. Early administration of acyclovir based on comprehensive clinical judgment effectively improved the clinical outcomes of children. These findings provide an important basis for the precise screening and comprehensive management of HSV in pediatric febrile cases. Notably, HSV can have asymptomatic recurrence, and positive test results do not mean that HSV is the only or direct etiological factor of fever. The occurrence of febrile symptoms in children is the result of the synergistic effect of HSV, co-detected pathogens and the immature host immune system, which should be fully considered in clinical diagnosis and treatment.

### 4.1 Epidemiological and clinical characteristics of HSV detected in febrile children

Our data indicated that children aged 1∼<3 years accounted for the highest proportion (56.0%) of HSV detected cases, which is consistent with domestic and international studies (5, 6), likely related to the ongoing development of the immune system and the decline of maternal antibody levels in this age group, making them more susceptible to HSV exposure and more likely to present with febrile symptoms. All enrolled children were healthy without any underlying diseases or comorbidities, which ruled out the influence of immune dysfunction or mucosal barrier damage on clinical manifestations, and confirmed that the observed symptoms were solely caused by HSV infection and its co-infections.

Notably, only 3.0% of children in this study presented with typical skin herpes, which is considerably lower than reports from some previous studies (7), while oral mucosal lesions (19.0%) and high fever (72.0%) were more prevalent. This non-specific clinical presentation is an important reason for the clinical diagnostic dilemma of HSV in febrile children, and also suggests that the clinical manifestations of HSV in children are diverse and cannot be judged only by typical skin lesions.The 4 cases with pertussis co-infection presented with typical pertussis-like cough, which reminded clinicians to pay attention to the superposition of symptoms caused by co-infections and avoid missed diagnosis of co-pathogens.

Therefore, for children with unexplained fever, especially those accompanied by oral mucosal symptoms, pharyngeal congestion and cervical lymphadenopathy, HSV should be included in the routine differential diagnosis, and timely combined serological and molecular biological testing should be performed to avoid misdiagnosis or underdiagnosis (8). The seizure rate of 5.0% also alerts clinicians to be vigilant about the neurotropic nature of HSV and the risk of central nervous system involvement (9). In this study, double positive results of HSV-IgM and HSV-DNA were considered as the main evidence of active HSV exposure, while single positive results were comprehensively judged in combination with clinical manifestations and co-detected pathogen status, which could effectively reduce the misdiagnosis rate caused by asymptomatic HSV recurrence. Studies have shown that the combination of HSV-IgM antibody and PCR detection can improve the screening efficiency of HSV in children, especially for hospitalized children with complete clinical samples (3, 6).

### 4.2 Clinical significance and mechanistic insights into the high rate of co-detected pathogens

A prominent finding of this study was the high co-detection rate of other pathogens (52.0%) in children with HSV detected,all of which were diagnosed by standardized pathogen-specific serological, PCR and microbial culture methods to ensure the accuracy of co-infection diagnosis.EBV was the most significant co-detected pathogen (27.0%), followed by Streptococcus pyogenes (6.0%) and Bordetella pertussis (4.0%).This phenomenon holds important clinical implications for the diagnosis and treatment of febrile children with HSV detected. The potential mechanisms for the high co-detection rate may include two aspects: (1) Immune Interference: HSV exposure can cause transient cellular immunosuppression in children with immature immune systems, creating conditions for the reactivation of latent viruses such as EBV (10). (2) Mucosal Barrier Disruption: Oral mucosal ulcers caused by HSV damage the body's physical mucosal barrier, potentially increasing the opportunity for other pathogens to invade, which might be an important route for bacterial co-detections (14.0%) (11).

This high co-detection rate also reminds clinicians that the detection of HSV in febrile children should not be regarded as a single final diagnosis, but should serve as an “alert” to proactively screen for other co-detected pathogens such as EBV and Bordetella pertussis using standardized detection methods. Comprehensive analysis of the results of HSV detection and co-detected pathogens is crucial for explaining the severity of the febrile symptoms, guiding the formulation of comprehensive treatment plans (e.g., whether antibacterial therapy or anti-EBV treatment is needed), and predicting the clinical prognosis of children.

### 4.3 Risk factors for liver impairment and optimization of treatment strategies

In this study, 30.0% of the children with HSV detected exhibited elevated transaminases, and intergroup comparisons indicated that EBV co-detection was an important factor associated with liver function impairment (*P* < 0.05). This suggests that liver injury in children with HSV detected is not likely to be the direct effect of HSV alone, but more likely the result of the synergistic effects of multiple pathogens or the systemic immune inflammatory response induced by pathogen exposure. Consequently, for children with HSV and EBV co-detected, enhanced dynamic monitoring of liver function should be part of the routine management strategy (12), and timely symptomatic liver protection treatment should be given once abnormal liver function is found.

Regarding treatment, this study confirmed the efficacy and safety of acyclovir in the clinical management of febrile children with HSV detected. A finding with greater clinical guiding value was that early antiviral treatment (within 24 h of admission) significantly shortened the time to defervescence and the duration of hospitalization (*P* < 0.001) (6), providing strong real-world evidence supporting the “early and adequate” use of acyclovir in clinical practice (13). It should be emphasized that the initiation of early antiviral treatment in this study was not based on HSV positive results alone, but on the comprehensive clinical judgment of three aspects: (1) high clinical suspicion of HSV-related febrile symptoms (typical manifestations such as high fever combined with oral mucosal lesions); (2) rapid etiological screening results (including HSV-IgM preliminary screening); (3) no other clear etiological factors found in the initial screening. This is in line with the practical principle of “treatment based on clinical judgment before definitive diagnosis” in pediatric emergency (6). The feasibility of early treatment was supported by the rapid PCR detection system used in our hospital, which provides results within 3–6 h, laying a foundation for the early initiation of targeted treatment.

Ninety-two children received intravenous acyclovir, which was determined based on the actual clinical status of the pediatric population: the study population was mainly infants and young children with a median age of 2.1 years, most of whom had oral mucosal lesions and persistent high fever, resulting in poor oral tolerance and inability to ensure the effective intake of oral drugs; intravenous acyclovir can ensure the rapid achievement of effective blood drug concentration and exert the antiviral effect in time.For the 8 children with mild symptoms, good oral tolerance and no obvious oral mucosal lesions, oral acyclovir was used for treatment, and all achieved good curative effects, indicating that the administration route of acyclovir should be individualized according to the clinical status of children.

Although acyclovir has a good safety profile in this study, given its renal excretion characteristics and potential nephrotoxic risk, clinical administration should still follow the principle of individualization, paying attention to hydration and intravenous infusion rate, especially for children with pre-existing renal or hepatic conditions (14). At the same time, for febrile children with HSV detected and high co-detection rate of other pathogens, antiviral treatment targeting HSV should be combined with targeted treatment for co-detected pathogens (such as antibacterial therapy for bacterial co-detection, liver protection treatment for abnormal liver function), and the treatment plan should be dynamically adjusted according to the changes of children's clinical symptoms and laboratory indicators, instead of only focusing on anti-HSV treatment.

### 4.4 Study limitations and future directions

This study has several limitations: (1) As a single-center retrospective study, selection bias is inevitable, and the research results need to be verified by multi-center prospective studies; (2) HSV-1/HSV-2 typing was not performed, preventing exploration of the correlation between different HSV types and clinical manifestations/severity—this is a notable limitation especially for infants under 1 year old, in whom HSV-2 exposure cannot be ruled out; (3) The lack of viral load data makes it difficult to establish a correlation between viral load and the severity of febrile symptoms; (4) Herpes simplex virus (HSV) DNA detection in cerebrospinal fluid (CSF) samples was not performed. It is well recognized that the detection of HSV DNA in CSF by polymerase chain reaction (PCR) is critical for the early and definitive diagnosis of herpes simplex encephalitis (HSE) in children (9). The absence of CSF PCR data may have led to an underestimation of the actual incidence of central nervous system involvement in this cohort. Future studies should routinely include CSF HSV PCR testing for children with HSV detected accompanied by neurological symptoms, to improve the diagnostic accuracy and comprehensive evaluation of disease severity; (5) Serological detection of HSV-IgG antibody was not performed, which prevents the differentiation between primary and recurrent HSV exposure and limits the in-depth analysis of the correlation between exposure type and clinical phenotypes or prognosis; (6) Due to the retrospective study design, the long-term prognosis of children with HSV detected and co-detected pathogens cannot be followed up, and the long-term effects of early acyclovir treatment need to be further confirmed.

Future research should develop in the direction of multi-center, prospective studies incorporating HSV typing and viral load quantitative detection, and should explore the role of host immune responses in the occurrence of febrile symptoms in children with HSV detected. At the same time, long-term follow-up of the enrolled children should be conducted to clarify the long-term clinical prognosis of children with HSV detected and co-detected pathogens. In addition, it is necessary to further study the clinical characteristics and treatment strategies of different types of HSV (HSV-1/HSV-2) in pediatric populations, so as to provide higher-level evidence for early warning of severe HSV-related diseases and individualized clinical treatment in children.

## Data Availability

The original contributions presented in the study are included in the article/supplementary material, further inquiries can be directed to the corresponding author.
